# Effect of body stiffness distribution on larval fish–like efficient undulatory swimming

**DOI:** 10.1126/sciadv.abf7364

**Published:** 2021-05-05

**Authors:** Tianlu Wang, Ziyu Ren, Wenqi Hu, Mingtong Li, Metin Sitti

**Affiliations:** 1 Physical Intelligence Department, Max Planck Institute for Intelligent Systems, 70569 Stuttgart, Germany.; 2 Institute for Biomedical Engineering, ETH Zurich, 8092 Zurich, Switzerland.; 3 School of Medicine and College of Engineering, Koç University, 34450 Istanbul, Turkey.

## Abstract

Energy-efficient propulsion is a critical design target for robotic swimmers. Although previous studies have pointed out the importance of nonuniform body bending stiffness distribution (*k*) in improving the undulatory swimming efficiency of adult fish–like robots in the inertial flow regime, whether such an elastic mechanism is beneficial in the intermediate flow regime remains elusive. Hence, we develop a class of untethered soft milliswimmers consisting of a magnetic composite head and a passive elastic body with different *k*. These robots realize larval zebrafish–like undulatory swimming at the same scale. Investigations reveal that uniform *k* and high swimming frequency (60 to 100 Hz) are favorable to improve their efficiency. A shape memory polymer–based milliswimmer with tunable *k* on the fly confirms such findings. Such acquired knowledge can guide the design of energy-efficient leading edge–driven soft undulatory milliswimmers for future environmental and biomedical applications in the same flow regime.

## Teaser

Larval fish–like soft robots reveal the effect of stiffness distribution on efficient swimming in the intermediate flow regime.

## INTRODUCTION

A large number of fish species use undulatory swimming for energy-efficient and fast locomotion (*1*). The traveling waves along their compliant body are generated by fluid-structure interaction and largely dictate their propulsion performance (*2*–*4*). Among all the factors affecting such interactions, the body bending stiffness distribution, *k*, plays a critical role (*5*, *6*). It is influenced by the body’s physical properties, such as the structural and material composition and active muscle modulation, i.e., stiffening the body by doing negative work (*3*, *6*, *7*). Most adult fish bodies in the inertial flow regime [i.e., the Reynolds number (*Re*) ≳ 2000] are known for their nonuniform *k* in both structure and material, where *k* decreases along the fish’s anterior-posterior axis (*5*, *6*, *8*). Previous robotic models or simulations emulating such nonuniformity have pointed out that the body with a nonuniform *k* could generate larger thrust and achieve higher energy efficiency than the uniform one. In these studies, researchers realize the nonuniformity by, e.g., adopting a geometry variation (*9*, *10*) or changing Young’s modulus of the material (*11*) along the body. While the benefits of nonuniform *k* have been investigated extensively in inertial undulatory swimmers, whether the swimmers in the intermediate flow regime (1 ≲ *Re* ≲ 2000), which have potential high-impact medical (*12*, *13*) and environmental (*14*, *15*) applications, could use the same mechanism to enhance swimming energy efficiency (*14*, *16*) remains elusive. Notably, because of the changing dominance of viscous forces and inertial forces in different regimes (*14*, *17*), the fluid-structure interaction in the intermediate flow regime is distinct from the ones in the inertial flow, and it might require a different role of *k* in improving energy efficiency.

To address such a challenge, we propose a class of untethered undulatory soft milliswimmers with uniform and nonuniform *k* to experimentally study the effects of different bioinspired *k* on their swimming energy efficiency. Particularly, these milliswimmers are inspired by larval zebrafish (*Danio rerio*). It has a continuous fin fold along its elongated body, typical among all teleost larvae in the intermediate flow regime (*18*). These robots can capture several key features of larval zebrafish in morphology, material properties, kinematics, and dynamics. Our investigations show that increasing swimming frequency from 30 to 100 Hz decreases the cost of transport (CoT) of the design with uniform *k*, while a similar benefit cannot be observed from the nonuniform cases. At high frequencies (60 to 100 Hz), the uniform design causes lower CoT than the nonuniform designs. Kinematics analyses reveal that the uniform *k* can generate improved body waveforms at high frequencies, which refines the wake flow pattern through increasing the fluid circulation and decreasing the jet angle, visualized by particle imaging velocimetry (PIV). To verify these findings further, we build a robotic larval fish with an on-the-fly, temperature-based tunable *k* by using a shape memory polymer (SMP) material (*19*). The test results demonstrate a sharp increase in the robot’s ability to swim against the water flow when it switches from the nonuniform *k* to the uniform one at high frequencies.

Given their miniature size and untethered design, these wireless mobile robots can be potentially used in future medical applications, such as targeted drug and other cargo (e.g., stem cells, genes, and imaging contrast agents) delivery inside the human body’s organs or structures filled with fluid (*13*). Fish-like robots adopting other on-board soft actuation methods at the similar time and length scale could also use the knowledge gained in this work to achieve energy-efficient swimming for environmental monitoring and remediation applications (*20*).

## RESULTS

### Soft milliswimmer design

**Fig. 1:**
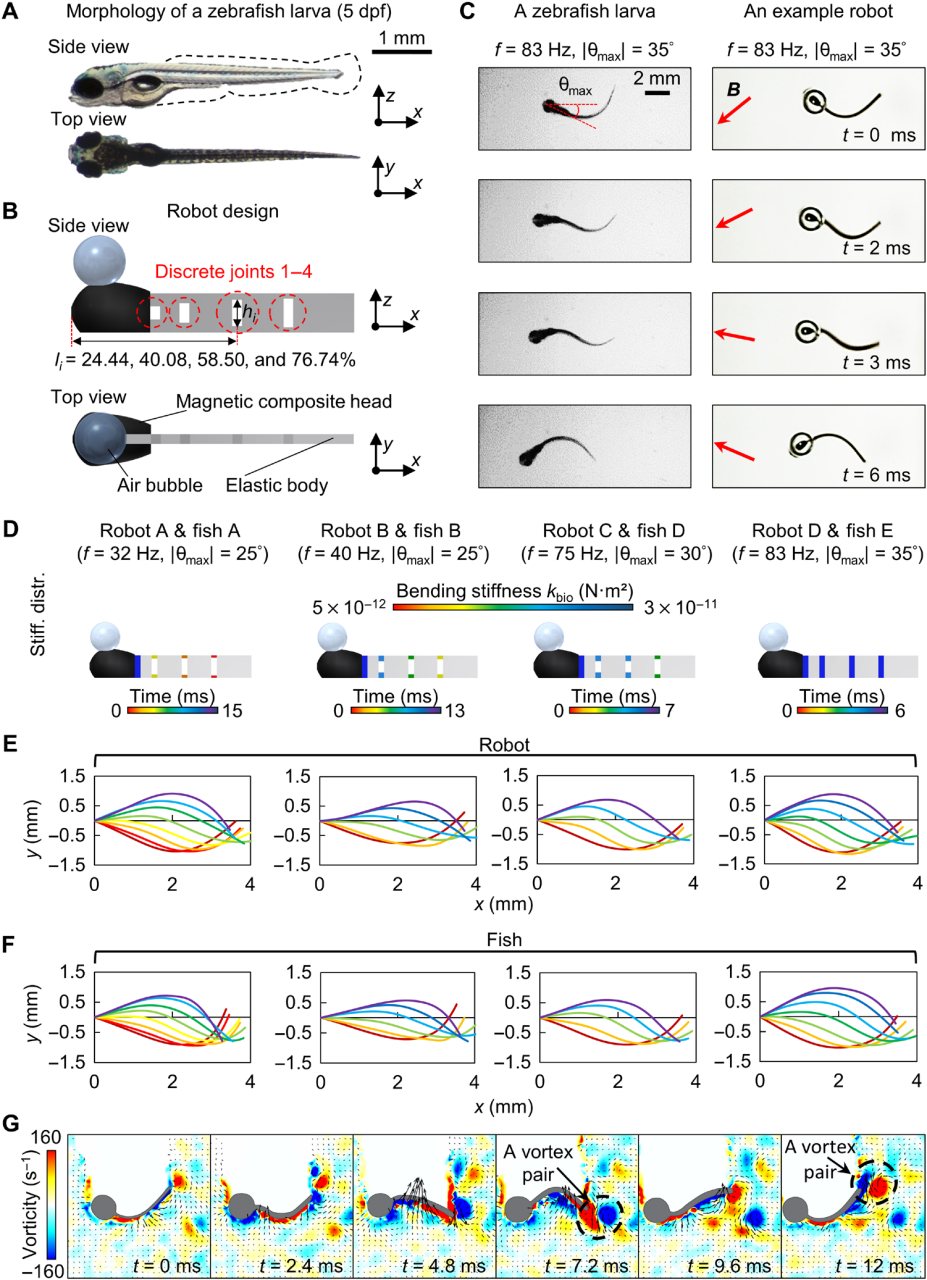
Larval zebrafish–like, untethered undulatory soft millirobot design. (**A** and **B**) Morphology of a zebrafish larva at 5 dpf [reproduced with permission from the Busch Lab: https://buschlab.org/resources/ and the work of Kettleborough *et al.* (*43*)] and robot design. Three major morphological properties of larval zebrafish at 2 to 5 dpf: overall dimension of 3.5 to 4.3 mm (*16*), relatively more uniform body shape than adult fish (*14*, *18*), and the location of the CoM at 29% of the total length (*21*) at the rest state. The design captures these features. The actuation is arranged at the leading edge (*6*, *10*). The geometry and material (Ecoflex 00-30) enable a similar order of *k* at 10^−11^ N·m as the organism (*21*, *22*). A uniform oscillating magnetic field controls the magnetic composite head, including swimming frequency, *f*, and amplitude of head pitch motion, |θ_max_|. The dimension of four discrete joint *h_i_* can be adjusted to emulate the global *k*. (**C**) A comparison of kinematics between a fish and a robot in half period during near-cyclic swimming. The orientation of the magnetic flux density is indicated. (**D** to **F**) Designs of bioinspired robots [robots A to D in (D)] to capture the biological midline kinematics (E and F). The frame rates for visualization are 500, 375, 750, and 1000 frames per second (fps). The metric to evaluate the emulation, the average error of sweeping areas, is controlled to be less than 5%. All biological data are reproduced from the work of van Leeuwen *et al.* (*21*). (**G**) PIV investigation of the bioinspired swimming in (C). Similar wake flow patterns as a zebrafish larva have been captured as two pairs of vortices are shed from the posterior part of the body per tail-beat cycle (*34*).

Inspired by the morphological properties of a larval zebrafish at 2 to 5 days post-fertilization (dpf) (*16*), we design the overall size of the soft milliswimmers to be 4.3 mm by 0.6 mm (length by width). Moreover, their body is designed to be rectangular shaped since the continuous median fin fold of larval zebrafish has not been replaced by separate fins (Fig. 1A) (*18*). Their center of mass (CoM) at the rest state is arranged to be 24 to 29% of the total body length from the snout tip, consistent with the organisms (*21*).

To isolate the effects of body bending stiffness distribution, *k*, while eliminating other influential factors, such as the muscle contraction doing positive work during swimming (*3*), we design an elastic body that can bend passively under the hydrodynamic load. The material is chosen to be Ecoflex 00-30 [Young’s modulus (*E*) = 0.125 MPa, Smooth-On Inc.], as it enables the robot to achieve a bending stiffness on the order of 10^−11^ N·m^2^, which is consistent with the organism, calculated on the basis of the biological data provided by van Leeuwen and colleagues (*21*, *22*). Given the body bending moment and curvature being on the order of 10^−8^ N·m and 10^−3^ m^−1^, respectively, *k* is estimated to be on the order of 10^−11^ N·m^2^.

To produce a caudally directed wave for propulsion, we impose a pitch motion at the robot’s head. Specifically, we arrange an active magnetic composite head at the elastic body’s leading edge (see the “Fabrication of magnetic composite head and elastic soft robot body” section in Materials and Methods). The time-varying torque is applied to the head by projecting a homogeneous oscillating magnetic field using two pairs of Helmholtz electromagnetic coils. Particularly, the swimming frequency *f* and the amplitude of head pitch motion, |θ_max_|, can be precisely controlled (see the “Magnetic actuation system and experimental setup” section in Materials and Methods). Thus, the robot head can achieve a pitch motion that exactly mimics its biological counterpart (*21*). Such a design scheme of imposing an active actuation source at the leading edge of an elastic foil aligns with the previous studies that replicate fish-like body kinematics at large length scales (*6*, *23*). Although some studies have pointed out the heave motion at the leading edge being also critical to realize fish-like undulatory swimming (*10*, *24*, *25*), the CoM oscillation in the lateral direction during larval fish near-cyclic swimming is negligible (*21*). Thus, we only focus on using the head pitch motion to induce undulatory swimming. Compared with other actuators such as electrical motors (*6*, *10*, *24*) and pneumatic actuators (*26*), external magnetic fields enable untethered actuation, allowing undisturbed swimming behavior and flow field for quantitative investigation. It also eases the robot’s miniaturization to the larval fish length scale and enables high swimming frequencies (up to 120 Hz in our current setup) at such length scales. To make the robot neutrally buoyant for easier data acquisition and analyses, we place an air bubble with a volume of 0.7 μl on top of the head using a pipette to reduce the robot’s effective density to around 1.08 g cm^−3^.

To practically emulate the global behaviors of changing *k*, we design four discrete joints along with the robot with their locations at 24.44, 40.08, 58.50, and 76.74% from the snout tip (Fig. 1B). The bending stiffness of each joint, *k*(*l_i_*) = *EI*(*l_i_*), can be fine-tuned by changing the second moment of area (*27*), *I*(*l_i_*), to mimic the midline kinematics of the organism. The fine-tuning goal is to minimize the average error of sweeping areas between the robot and the organism’s midlines in half of a cycle (denoted as “average error of sweeping areas”), represented by a percentage. Currently, we adopt 5% as a threshold for an acceptable emulation (see the “Design of discrete body joints for controlled body stiffness distributions” section in Materials and Methods).

To cover *f* within a biologically relevant range, we use four bioinspired designs—robots A, B, C, and D (with $k_{\text{bio}}^{A}$ to $k_{\text{bio}}^{D}$, respectively)—to emulate the midline kinematics of larval fish swimming at five different *f* [fish A, B, C, D, and E are reproduced from the work of van Leeuwen *et al.* (*21*); see the “Data selection for the range of undulatory kinematics” section in Materials and Methods], as shown in Fig. 1 (C to F). Robot C is found to be able to realize the midline kinematics of both fish C and fish D. The complete comparison of the midline kinematics is shown in fig. S3 and movie S1. An acceptable emulation of the midline kinematics using the given robot configurations confirms the critical role of *k* in the fluid-structure interaction in the intermediate flow regime.

In addition to the similarity in morphology, material property, and midline kinematics, the robot can also capture three key features of larval zebrafish swimming related to swimming dynamics: the Reynolds number (*Re*) range, the Strouhal number (*St*) range, and the wake flow patterns (Fig. 1G). Please refer to table S1 for detailed comparisons.

### Effects of body bending stiffness distribution on the energy efficiency of cyclic swimming

Three metrics—swimming speed ${\bar{\overset{\cdot }{w}}}_{x}$, input power *P*_in_, and CoT—are used to quantitatively evaluate the cyclic swimming of these robots under the conditions similar to the ones of experimental observations on the organism, i.e., near-cyclic swimming in still water (see the “Evaluation of the robot swimming performance” section in Materials and Methods) (*21*). CoT is chosen to evaluate the locomotion-related energy efficiency since it is relevant for assessing the cyclic swimming of fish and robotic fish models (*10*, *14*).

**Fig. 2:**
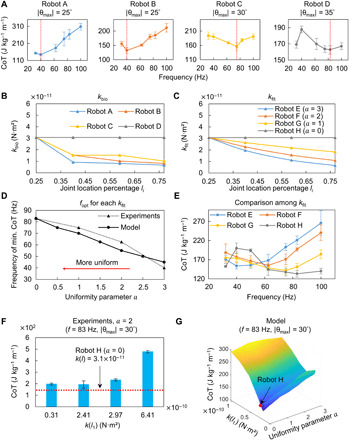
Effect of body bending stiffness distribution (*k*) on energy efficiency (CoT). (**A**) CoT of the robots as a function of frequency. Each |θ_max_| is kept to be the same as the individual biological counterpart from which *k*_bio_ is extracted. As an example in (i), $k_{\text{bio}}^{A}$ of robot A is extracted from fish A where *f* = 32 Hz and |θ_max_| = 25°. Thus, |θ_max_| = 25° is maintained for robot A. For each one among robots A to D, there is a turning frequency *f*_opt_ that leads to the improved CoT given that specific *k*_bio_ and |θ_max_|. Modeling results confirm this phenomenon (fig. S8). (**B** and **C**) Designs of *k*_bio_ and *k*_fit_. To quantify the bioinspired designs in (B) while enabling better-controlled comparison, in (C), we use exponential functions to fit the bioinspired designs by *k*(*l_i_*)_fit_ = *k*(*l*_1_)·exp(−*a*(*l_i_* − 0.24)), where *a* indicates the uniformity of the distribution. (**D** and **E**) Investigation of the exponential robots. The relation between body uniformity, parametrized by *a*, and *f*_opt_ (D). At higher *f* (60 to 100 Hz), the uniform design enables the noticeable energy efficiency improvement when compared with itself at lower frequencies and when compared with the other nonuniform designs at higher frequencies (E). (**F** and **G**) Exploration beyond the biologically relevant *k*_fit_. Experimental investigation on the stiffer nonuniform designs with *a* = 2 at 83 Hz (F). They demonstrate no capability in efficiency improvement compared with robot H, the biologically relevant uniform design. Further parameter space exploration around robot H using the dynamics model (G). Robot H is close to the design to achieve the local optimum of CoT at 83 Hz. Similar to the experimental results, stiffer nonuniform designs do not improve the efficiency. Error bars represent the SDs of the means and the number of trials *n* = 5.

We first analyze the CoT of four individual bioinspired robots (robots A to D with $k_{\text{bio}}^{A}$ to $k_{\text{bio}}^{D}$) at biologically relevant frequencies (30 to 100 Hz) (*21*). The amplitude of head pitch motion, |θ_max_|, of each robot emulates their corresponding biological counterparts, i.e., fish A, B, D, and E, which are 25°, 25°, 30°, and 35°, respectively (note that |θ_max_| is the same for fish A and B). The experiment results indicate that for each design with a specific *k*_bio_ and |θ_max_|, there is an optimal turning frequency, *f*_opt_, that leads to the optimal energy efficiency (Fig. 2A).

A close inspection of Fig. 2A reveals that *f*_opt_ shifts with varying *k*_bio_. To enable a well-controlled comparison, we design a group of robots whose *k*_fit_ satisfies exponential functions, i.e., *k*(*l_i_*)_fit_ = *k*(*l*_1_)·exp(−*a*(*l_i_* − 0.24)) with the uniformity parameter *a* = 3, 2, 1, and 0 (robots E, F, G, and H, referred to as exponential robots henceforth). These designs could quantify *k* uniformity through a single parameter, *a*, and cover the range of $k_{\text{bio}}^{A,B,C,D}$ while enabling more distinguishable *k* (Fig. 2, B and C). The design parameters of these exponential robots are summarized in table S3. |θ_max_| is controlled to be 30° throughout the frequency sweeping experiments (see movies S2 and S3). The experiment results indicate a negative correlation between *a* and *f*_opt_ (Fig. 2D). In other words, *f*_opt_ shifts from lower to higher values when *k*_fit_ of the body becomes more uniform. To explore the shift of *f*_opt_ in a larger searching space of *k*_fit_, we develop a computationally efficient dynamic model (see the “Dynamic modeling of the fluid-structure interaction” section in Materials and Methods). The model further confirms the shift rule of *f*_opt_ (Fig. 2D). A detailed comparison of CoT at different *f* among different *k*_fit_ (Fig. 2E) reveals that when increasing *f* from 40 to 83 Hz, the CoT of the uniform design gradually decreases and reaches the minimum at around 83 Hz. This CoT value is also the lowest that we can achieve within the group. While at lower *f* (30 to 60 Hz), the superiority of uniform or nonuniform *k*_fit_ on improving energy efficiency is inconclusive because of the overlapping of CoT profiles for different *k*_fit_ designs.

Although the advantage of the uniform *k* at high *f* has been demonstrated, it remains unclear whether an even stiffer design, which has *k* outside the biologically relevant range, with nonuniform distribution, could further improve the energy efficiency. Thus, without the loss of generality, we experimentally evaluate several designs with increased *k*(*l*_1_)_fit_ and *a* = 2 at 83 Hz (Fig. 2F). The variation in *k*(*l*_1_)_fit_ is enabled by different ratios of curing agents in the elastomer base for polydimethylsiloxane (*28*). The analyses of CoT indicate that only the biologically relevant uniform design with *k*(*l*)_fit_ = 3.1 × 10^−11^ N·m^2^, i.e., robot H, leads to the best CoT. Furthermore, we adopt the dynamics model to explore the parameter space around the design of robot H. The results are consistent with the above experiment evidence that robot H is around the design to achieve a local minimum of CoT, as shown in Fig. 2G.

Since all of the above investigations study the effect of *k* on energy efficiency by sweeping *f* when |θ_max_| is fixed, as a supplement, we carry out the experiments by sweeping |θ_max_| while fixing *f*. The results indicate that the speed, input power, and CoT almost linearly increase with the increasing |θ_max_|. There is no turning |θ_max_| within the biologically relevant range (8° to 42.5°) that makes CoT reach the local minimum (fig. S10). Moreover, we relax the constraints on *k*_bio_ and test the robots with modified distribution patterns *I*(*l*), e.g., softer anterior and stiffer posterior. These designs cannot realize the larval zebrafish–like midline kinematics, and they underperform the bioinspired designs (robots A to D) in terms of energy efficiency (fig. S11).

### Analyses of undulatory swimming kinematics and wake flow patterns

**Fig. 3:**
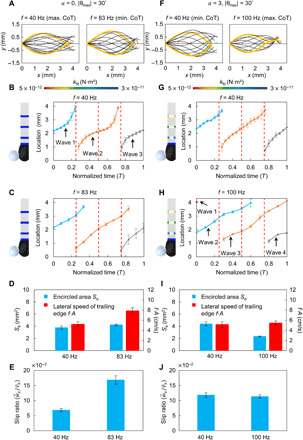
Analyses of the robot’s undulatory swimming kinematics. (**A** to **E**) Analyses of the uniform *k* design (*a* = 0) swimming at two *f* corresponding to reaching maximal and minimal CoT. Midlines for 40 and 83 Hz in 1 cycle are shown with 1000 fps (A). The transmissions of a body wave from head to tail are both finished around 0.5*T*, where *T* denotes the period (B and C). Increasing *f* raises the encircled area *S*_e_ by 12.3% and lateral velocity of the trailing edge *fA* by 50.8% (D). With differences in waveforms, the average swimming speed ${\bar{\overset{\cdot }{w}}}_{x}$ is increased by 248.3% (fig. S9) and the slip ratio ${\bar{\overset{\cdot }{w}}}_{x}/{\bar{v}}_{x}$ is increased by 146.4% (E), where ${\bar{v}}_{x}$ represents the average propulsive wave speed. Since the input power *P*_in_ is increased by 131.2% (fig. S9), consequently, CoT is decreased by 33.3%. (**F** to **J**) Analyses of the nonuniform *k* design (*a* = 3). Midlines for 40 and 100 Hz in 1 cycle are shown with 1000 fps (F). Increasing *f* slows down the transmission of body wave from 0.5*T* to 0.75*T* (G and H). It decreases *S*_e_ by 47.0%, whereas *fA* does not vary much (*t* test, *P* = 0.3281) (I). The deteriorated waveforms lead to ${\bar{\overset{\cdot }{w}}}_{x}$ improved by 48.8% (fig. S9), and ${\bar{\overset{\cdot }{w}}}_{x}/{\bar{v}}_{x}$ does not change significantly (*P* = 0.2940) when *f* is increased (J). Since *P*_in_ is increased by 160.4% (fig. S9), consequently, CoT is increased by 73.0%. The resultant CoT is much higher (increased by 49.9%) than that of the uniform *k* design achieved at high *f*. Error bars represent the SDs of the means and the number of trials *n* = 5.

Without the loss of generality, we analyze the uniform *k* design (*a* = 0) and the most nonuniform *k* design (*a* = 3) swimming at *f* where the CoT reaches the maximum and the minimum, respectively. The midline profiles and body waves passing from the head to tail in 1 cycle, *T*, are shown in Fig. 3 (A to C and F to H). In practice, body waves can be identified by detecting the turning points of the midline profile of each time step. While it takes around 0.5*T* to transmit a body wave from head to tail for the rest cases, it takes nearly 0.75*T* for the nonuniform design swimming at 100 Hz. To quantitatively analyze the differences of the kinematics among four cases, we use three specific metrics, i.e., encircled area by extreme midlines *S*_e_, lateral speed of the trailing edge *fA*, and the slip ratio ${\bar{\overset{\cdot }{w}}}_{x}/{\bar{v}}_{x}$, where *A* represents peak-to-peak tail amplitude, ${\bar{\overset{\cdot }{w}}}_{x}$ represents the average swimming speed, and ${\bar{v}}_{x}$ represents the average propulsive wave speed. *S*_e_ reflects the thrust generation since it is directly related to the magnitude of the body’s lateral excursions. Increasing the magnitude of the body’s lateral excursions leads to the increased amount of fluid entrained and accelerated along the body, which, in turn, increases the fluidic forces produced (*10*). *fA* can evaluate the thrust, which approximately scales with (*fA*)^2^, as suggested by Voesenek *et al.* (*14*). ${\bar{\overset{\cdot }{w}}}_{x}/{\bar{v}}_{x}$ can be used to indicate the energy efficiency of undulatory swimmers (*29*), and a larger value often suggests that more momentum of water is pushed backward effectively (*30*). In summary, achieving higher *S*_e_, *fA*, and ${\bar{\overset{\cdot }{w}}}_{x}/{\bar{v}}_{x}$ with lower input power *P*_in_ indicates better waveforms in terms of energy efficiency.

For the uniform *k* design (*a* = 0), increasing *f* from 40 to 83 Hz raises the *S*_e_ and *fA* by 12.3% (*t* test, *P* = 9.7 × 10^−3^) and 50.8% (*t* test, *P* = 5.4 × 10^−5^), respectively (Fig. 3D). With such differences in waveforms,$\;{\bar{\overset{\cdot }{w}}}_{x}$ is increased by 248.3% (*t* test, *P* = 1.07 × 10^−9^) (fig. S9) and ${\bar{\overset{\cdot }{w}}}_{x}/{\bar{v}}_{x}$ is increased by 146.4% (*t* test, *P* = 4.3 × 10^−7^) (Fig. 3E). Since *P*_in_ is increased by 131.2% (*t* test, *P* = 2.3 × 10^−9^) (fig. S9), consequently, CoT is decreased by 33.3% (*t* test, *P* = 1.6 × 10^−5^).

For the most nonuniform *k* design (*a* = 3), increasing *f* from 40 to 100 Hz decreases *S*_e_ by 47.0% (*t* test, *P* = 1.3 × 10^−6^), whereas *fA* does not vary much (*t* test, *P* = 0.3281) (Fig. 3I). The deterioration of the waveforms leads to ${\bar{\overset{\cdot }{w}}}_{x}$ improved only by 48.8% (*t* test, *P* = 1.9 × 10^−8^) (fig. S9). ${\bar{\overset{\cdot }{w}}}_{x}/{\bar{v}}_{x}$ does not change significantly (*t* test, *P* = 0.2940) when *f* is increased (Fig. 3J). Since *P*_in_ is increased by 160.4% (*t* test, *P* = 1.8 × 10^−9^) (fig. S9), consequently, the CoT is increased by 73.0% (*t* test, *P* = 6.5 × 10^−5^). The resultant CoT is much higher (increased by 49.9%; *t* test, *P* = 2.1 × 10^−5^) than that of the uniform design achieved at high *f*.

**Fig. 4:**
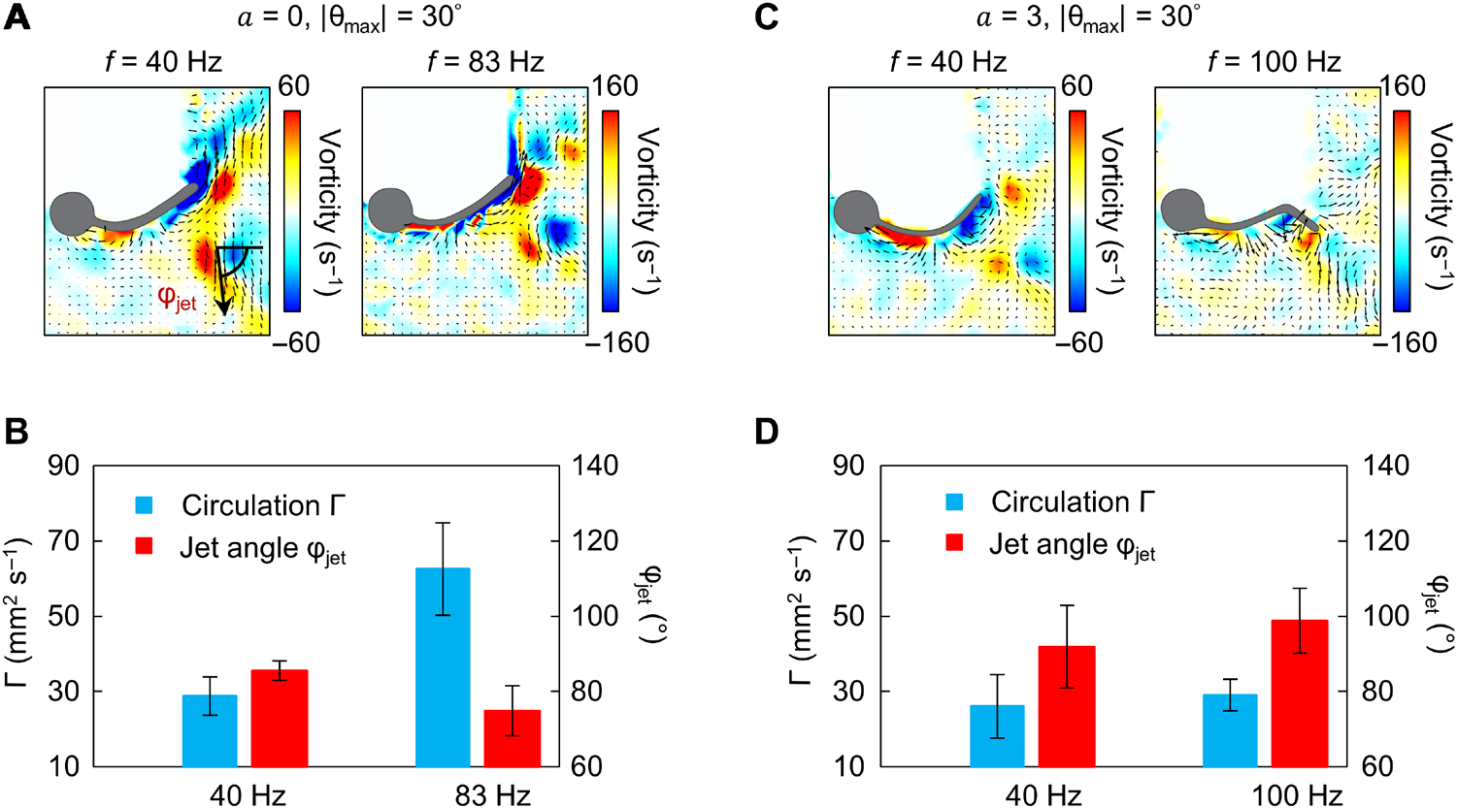
Analyses of the wake flow patterns for the uniform and nonuniform *k* designs at different swimming frequencies. (**A** and **B**) Analyses of the uniform *k* design (*a* = 0) swimming at 40 Hz (maximal CoT) and 83 Hz (minimal CoT). In (A), the vorticity field (color map) and velocity vector field (black arrows) are adjacent to the robots. Increasing *f* raises circulation Γ by 117.1% (*t* test, *P* = 4.6 × 10^−4^) and decreases the jet angle φ_jet_ by 12.4% (*t* test, *P* = 0.01). (**C** and **D**) Analyses of the nonuniform *k* design (*a* = 3) swimming at 40 Hz (minimal CoT) and 100 Hz (maximal CoT). In (C), the vorticity field (color map) and velocity vector field (black arrows) are adjacent to the robots. The increase in *f* from 40 to 100 Hz does not significantly influence Γ and φ_jet_ (*t* test, *P* = 0.5 and *P* = 0.3, respectively). When comparing the uniform and nonuniform *k* designs both at high *f*, Γ of the uniform design is 115.6% (*t* test, *P* = 4.2 × 10^−4^) larger than the one of the nonuniform design when φ_jet_ is 24.2% (*t* test, *P* = 1 × 10^−3^) smaller. The refinement in wake flow patterns demonstrates that increasing *f* could benefit the uniform body. Error bars represent the SDs of the means and the number of trials *n* = 5.

To investigate how the variations in undulatory swimming kinematics affect the wake flow patterns, we implement PIV experiments (see the “PIV system” section in Materials and Methods) for the above designs (Fig. 4). Two metrics are used to evaluate the wake structures: the fluidic circulation, Γ, and the jet angle, φ_jet_. Γ represents the vortex strength, which is proportional to the propulsion force. It is calculated by integrating the vorticity over the area occupied by the vortex core (*31*). Γ of a vortex pair is estimated by averaging the circulation of the clockwise and the counterclockwise vortices. φ_jet_, as labeled in Fig. 4A, defines the direction of the shooting flow between two vortices. A smaller φ_jet_ suggests that the shooting flow is directed more parallel to the propulsion direction, which is favorable for increasing the swimming efficiency since less momentum of water is transferred laterally (*31*). The quantitative analyses of Γ and φ_jet_ provide two important observations. First, for the uniform *k* design, φ_jet_ is decreased by 12.4% (*t* test, *P* = 0.01), and Γ is increased by 117.1% (*t* test, *P* = 4.6 × 10^−4^) when *f* increases. For the nonuniform *k* design, neither of these happens (*t* test, *P* = 0.3 and *P* = 0.5, respectively). Second, when comparing the uniform and nonuniform designs at high *f*, the uniform design is obviously superior in improving both Γ and φ_jet_. At high *f*, Γ of the uniform design is 115.6% (*t* test, *P* = 4.2 × 10^−4^) larger than the nonuniform *k* design, while φ_jet_ is 24.2% (*t* test, *P* = 1 × 10^−3^) smaller. The refinement in wake flow patterns further demonstrates that increasing *f* could benefit the uniform body. The details of the direct comparison between the designs of *a* = 0 and *a* = 3 at lower frequencies and higher frequencies can be found in note S7.

### Stiffness distribution–tunable soft robotic undulatory swimming

**Fig. 5:**
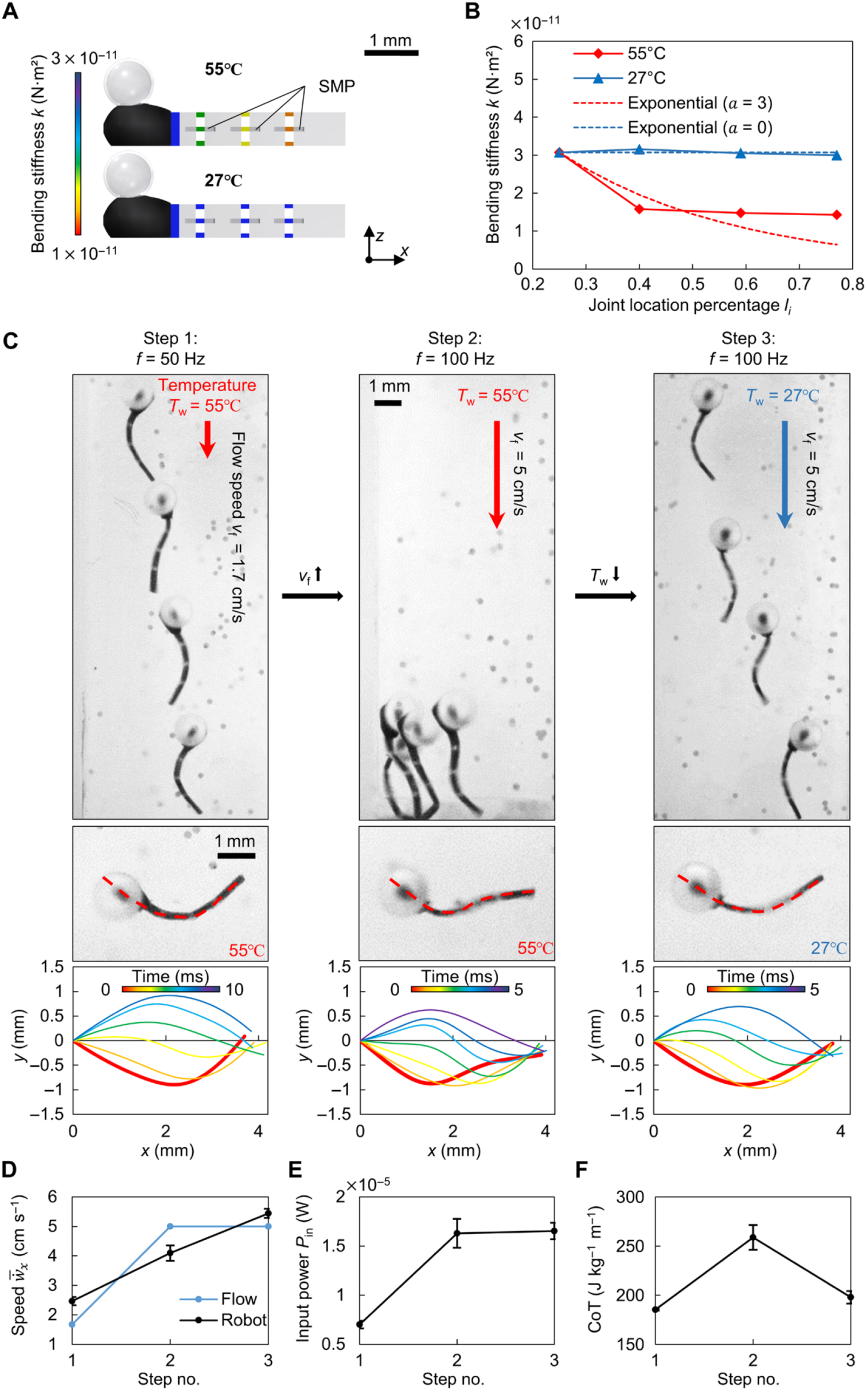
Design and demonstration of the soft milliswimmer with adjustable *k* using SMP-integrated joints that can change their elastic modulus drastically by temperature increase. (**A**) The *k*-adjustable robot comprises a magnetic composite head, an elastic body, and SMP films attached to the joints. *k* is adjusted by varying the temperature of its swimming environment. (**B**) *k* of the robot at two temperatures. *k* at 55°C can be approximated by the exponential distribution *k*_fit_ with *a* = 3, corresponding to the *f*_opt_ at around 50 Hz. *k* at 27°C can be approximated by *a* = 0, corresponding to *f*_opt_ at around 100 Hz (Fig. 2D). (**C**) Demonstration of against-flow swimming and midline kinematics comparison. |θ_max_| is kept as 30°. At 55°C with *a* = 3, the robot could swim against the flow efficiently at *f*_opt_ = 50 Hz (step 1). When the flow speed is increased while *k* is not changed, increasing *f* to 100 Hz alone cannot lead to efficient swimming, and the robot cannot swim against the flow (step 2). When the temperature is decreased to 27°C and *a* = 0, the robot can swim efficiently against the flow at around 100 Hz (step 3). The apparent deterioration of waveforms can be observed from step 2, compared with step 3. (**D** to **F**) Investigation of speed, input power, and CoT of the robot under static water conditions. While almost the same input power is applied in steps 2 and 3, the robot could only achieve a higher speed and lower CoT in step 3. Error bars represent the SDs of the means and the number of trials *n* = 5.

To visually verify the superiority of uniform *k* design in high-frequency propulsion, here, we endow the robot with the capability of changing its *k* on the fly by integrating SMP (*19*, *32*) to its joints (see the “Stiffness distribution–tunable SMP-based robot and its experimental setup” section in Materials and Methods). Because of the temperature-dependent drastic Young’s modulus variation of the SMP-integrated joints, the stiffness distribution along the robot can be adjusted by changing the temperature of the surrounding water. On the basis of this approach, we fabricate a robot with two distinct *k* at 27° and 55°C (Fig. 5, A and B): *k* at 27°C can be well approximated by *k*_fit_ with *a* = 0 (uniform *k*), while *k* at 55°C can be approximated by *k*_fit_ with *a* = 3 (nonuniform *k*). These two exponential *k* correspond to *f*_opt_ at around 83 Hz and 40 to 50 Hz, respectively, according to the results shown in Fig. 2D.

Next, we test the robot in a custom flow tank and let it swim against the incoming water flow. In all steps, |θ_max_| is strictly controlled to be 30°. In step 1 (55°C), the robot swims with *k*_fit_ parameterized by *a* = 3. According to Fig. 2D, efficient swimming based on this given *k*_fit_ can be achieved at around 50 Hz, and it can swim against the flow (step 1 in Fig. 5C) with the speed of 1.7 cm s^−1^. In step 2, the flow speed increases almost threefold to 5 cm s^−1^. Although *f* also increases to 100 Hz, the robot cannot swim efficiently because of the improper combination of *k*_fit_ with *a* = 3 and *f* (step 2 in Fig. 5C), and it cannot swim against the flow. In step 3, the cooling of the water converges the *k*_fit_ with *a* = 3 to *k*_fit_ with *a* = 0, and 100 Hz is close to *f*_opt_ of this distribution. The robot could now swim efficiently against the flow (step 3 in Fig. 5C). The complete procedure is demonstrated in movie S4.

The midline kinematics of three steps are extracted for comparison in Fig. 5C, consistent with the ones investigated above for exponential robots. The midlines of step 2 indicate deteriorated waveforms. Figure 5 (D to F) demonstrates the quantitative analyses of the swimming performance along with three steps. While almost the same input power is applied in steps 2 and 3, the robot could only achieve a higher speed and lower CoT in step 3. This is enabled by the proper combination of *k* and *f*_opt_, which confirms the importance of *k* in larval fish swimming. This experiment platform, with the capability of controlled flow circulation, can be used to investigate the swimming behaviors in complex dynamic flow conditions for future work.

Note that these verification experiments rely on temperature control to induce an on-the-fly tunable *k*. This is different from the previous systematic investigations, where the water temperatures are strictly monitored and controlled to ensure that the influences of viscosity changes can be minimized. To investigate how much the flow regime has varied when the temperatures are changed in the experiments, we compute the relevant *Re* (see note S2.2), which is 274 at 27°C and 207 at 55°C. Since both cases are in the intermediate flow regime, a reasonable comparison can be made.

## DISCUSSION

On the basis of the study of undulatory swimming of the soft milliswimmers proposed here, while each body stiffness distribution, *k*, corresponds to a unique optimal swimming frequency within the biologically relevant frequency range, the uniform *k* swimming at higher frequencies (60 to 100 Hz) significantly improves the energy efficiency, i.e., decreasing the CoT, in the intermediate flow regime (1 ≲ *Re* ≲ 2000). The kinematics analyses attribute this refinement to the improvement of the body wave qualities, which increases the circulation and decreases the jet angle of the wake flow. We also demonstrate in movie S5 that the external magnetic field can steer such soft robotic fish locomotion. The miniature robot design can be used for potential future medical (e.g., targeted cargo delivery) applications inside the fluid-filled regions of the human body. Furthermore, the undulatory swimming kinematics with the energetically beneficial elastic mechanism shown in this study can also be achieved by other miniature on-board actuation methods at the similar *Re*. Such on-board actuated and powered swimmers would have other potential applications requiring energy-efficient locomotion, such as biological species monitoring and environmental remediation.

Although the nonuniform stiffness distributions have been extracted from the emulation of midline kinematics of larval zebrafish, these distribution patterns seem not as effective as the uniform one to assist the energy-efficient cyclic swimming under the current test conditions. However, its role in other swimming behaviors, such as maneuvers, requires further investigation in the future. Relaxing the current static water conditions to the unsteady flows may offer more information to explain the function of the nonuniform *k* (*33*).

As one of the most widely spread locomotion modes, undulatory swimming exists in many organisms, ranging in size from sperm cells to whales (*34*). It results from the fluid-structure interaction, which involves the influence of the muscle contraction and the interactions between the fish body and the surrounding water. This can be shown by the mismatch between the body wave’s transmission speed and the muscle contraction’s propagation speed (*3*). Particularly, the axial muscle contraction can do both positive work to bend the body and negative work to stiffen the body (*6*, *7*). The negative work part and the body’s physical properties, i.e., structural and material properties, determine the body bending stiffness distribution, *k*, which dictates the fluid-structure interaction (*6*, *10*). Using the proposed robotic platform in this study, we abstract and emulate a series of *k* configurations of larval fish-like swimming at the steady state and investigate their effects. This robotic platform can be further extended to investigate the effects of different *k* on biological larval fish and illustrate the importance of negative work in real larval fish swimming. This could help explain the effectiveness of their propulsion with usually higher frequencies (*21*, *35*, *36*) and hint us to reexamine the roles of the homogeneously stiff notochord (*16*, *37*) and the uniform body morphology (*14*, *18*) on efficient undulatory swimming at the larval phase.

## MATERIALS AND METHODS

### Fabrication of magnetic composite head and elastic soft robot body

The overall dimension of the cube encapsulating the magnetic composite head is 1.2 mm by 0.8 mm by 0.8 mm (length by width by height). We design a concave shape on top of the head. A 10-μl pipette is used to place an air bubble into this concave shape to reduce the robot body’s overall density. Because of the hydrophobicity of the surface, the air bubble can be firmly trapped (fig. S4 and note S6). The head is a composite of the urethane rubber (Clear Flex 95, Smooth-On Inc.) and the neodymium-iron-boron (NdFeB) magnetic particles (MQP-15-7, Magnequench; average diameter, 5 μm) with a mass ratio of 1:4. The mixed material is cast into a negative mold made of soft silicone rubber (Mold Max 20, Smooth-On Inc.). This negative mold is previously made out of a positive mold, which is directly three-dimensional (3D) printed (Clear Resin BV-007 and PICO2, ASIGA). After casting, the negative mold is kept on a hot plate of 60°C curing for 1 hour. The cured magnetic composite head is 1.15 mg on average, with an average density of 2.77 g cm^−3^. To magnetize the heads, we put them inside a vibrating sample magnetometer (EZ7, MicroSense) and apply a strong uniform magnetic field with the magnitude of flux density of 1.8 T. The direction is from the end connecting the elastic body to the snout tip. This procedure leads to the effective volume magnetization of the head to be around 1.79 × 10^5^ A m^−1^. The complete procedures of head fabrication are shown in fig. S4 (A to D).

The elastic body is made of soft silicone rubber (Ecoflex 00-30, Smooth-On Inc.). A small amount of pigment (Silc Pig, Smooth-On Inc.) is mixed into the silicon rubber (mass ratio of 0.1:1) to assist laser cutting in the later procedures. The mixture is cast onto a flat poly(methyl methacrylate) (PMMA) plate coated with a thin layer of parylene C (6 μm thick), which is used to ease the release of the cured rubber. After curing for 1 hour on the hot plate at 60°C, a thin film with a thickness of around 170 μm is formed. The ultraviolet (UV) laser (ProtoLaser U3, LPKF Laser & Electronics AG) is used to cut the desired body designs out of the film. All bodies are with the overall dimension of 3.5 mm by 0.6 mm by 0.17 mm (length by width by height). The dimensions of the joints vary among different designs. The fabrication procedure of the body is shown in fig. S4E.

In the final step, the robot is assembled by inserting the leading edge of the body into the gap at the posterior part of the head. The soft silicon rubber (Ecoflex 00-10, Smooth-On Inc.) is used to glue this connection firmly. After curing on the hot plate of 60°C for another 5 min, the robot is ready for tests (fig. S4F).

### Magnetic actuation system and experimental setup

Two pairs of Helmholtz electromagnetic coils are used to provide homogeneous magnetic fields in 2D with the oscillating frequencies up to 120 Hz when the effective magnitude of the magnetic flux density is 2 mT, which is directly measured by a gaussmeter (Model 460 3-Channel Gaussmeter, Lake Shore Cryotronics Inc.) For all experiments, the magnetic flux density along the propulsion direction *B_x_*, is kept constant at −2 mT. The one perpendicular to the propulsion direction *B_y_* is adjusted to meet different amplitudes of head pitch motion |θ_max_| and swimming frequency *f*. An example of the actuation signal and the corresponding robot swimming is shown in fig. S5.

Experiments are carried out in a transparent water tank positioned at the central region of the coil system. The dimension of the tank is 100 mm by 60 mm by 40 mm (length by width by height). The robots are launched from a stage fixed at the bottom of the tank, whose location is slightly behind the center of the coil system. To minimize the influence of viscosity change due to the temperature variation and guarantee the comparability of experiment data, we monitor the water temperature by a thermocouple and control it to be at 23 ± 1.5°C for each trial. To reduce the effects of friction between the robots and the launch stage, we only use the images after the robots swim away from the stage for at least three periods. We also synchronize the trigger time of magnetic actuation and the recording of a high-speed camera (M310, Phantom Inc.) by a real-time control system (CompactRIO, National Instruments Corporation) for quantitative analyses of performances. The diagram of the experiment setup is shown in fig. S6. The experiment environment–related parameters are summarized in table S5. To further justify that the robot is actuated by magnetic torque and the induced undulation rather than magnetic force pulling, we show that a single oscillating magnetic composite head cannot propel itself (see movie S6).

### Design of discrete body joints for controlled body stiffness distributions

We adopt the following procedures first to extract midlines from the dataset of larval zebrafish swimming (*21*): (i) selection of points along the body midline from the snout tip to the tail tip; (ii) using the quadratic spline to interpolate the selected points with 51 evenly distributed points; (iii) using the Gaussian filter to smooth the curve; (iv) normalization of the curve length to 4.3 mm; and (v) aligning the snout tips of all the extracted midlines together to the origin of coordinate for the convenience of discretization, comparison, and kinematics analyses. Note that the same procedures are used to process the robot data from a high-speed camera (M310, Phantom Inc.) recording later.

To discretize the robot body, we use piecewise linear functions with five segments to fit the midlines extracted from a particular fish swimming case (fish B; table S7) in one near-cyclic period (fig. S2, A and B). For all fitted midlines, the locations of four inflection points from the snout tip, represented by percentages, are summarized in four histograms (fig. S2C). Note that the locations of the inflection points vary in a cycle. For example, the locations of the first inflection point in 1 cycle vary between 11 and 36%, with a majority between 24 and 30%. Gaussian distributions are then used to estimate the means of the inflection point location (fig. S2D). These values are regarded as the locations for four joints. To validate these joint locations, we compute the average error of sweeping areas between the biological midlines that we target at mimicking and the fitted five-segmentation piecewise linear functions using the inflection points indicated above. To compute these errors, we move the processed midlines to the location with snout tips at (0, 2) (unit, mm). The sweeping area of each midline is defined as the area encircled by the instant body midline and the *x* axis. For each time instance, the error of the sweeping area is defined as the deviation of the area for the fitted functions from the one for the organism midlines, represented by a percentage. It turns out that the errors for all cases are less than 1%, as shown in fig. S2E, which shows the applicability of such a joint arrangement. Hence, the joint locations are arranged with such four values for all robots (Fig. 1B). Note that the definition of the average error of sweeping areas also applies in the later emulation of biological midlines using our robotic models.

To tune the bending stiffness of four joints, we remove the proper amount of material by UV laser (ProtoLaser U3, LPKF Laser & Electronics AG) at each joint so that *I*(*l_i_*) is changed. This design approach leads to different dimensions of joint holes, which might cause the differences in water leakage through these holes and further affect the drag on the robots. However, on the basis of our experiment investigations, these differences do not lead to significant effects (note S1).

### Data selection for the range of undulatory kinematics

We make a complete survey on the dataset reproduced from the work of van Leeuwen *et al.* (*21*), which provides the kinematics of near-cyclic swimming of larval zebrafish at 2 to 5 dpf. The focus is on the range of swimming frequency *f* and amplitude of head pitch motion |θ_max_|. The statistics (fig. S1) show that within 38 groups, the fish larvae swim at *f* from 30.9 to 100 Hz, in which more than 81% cases are over 60 Hz. |θ_max_| ranges from 8° to 42.5°, with 50% cases ranging from 26° to 35°. To cover this range, we select five cases to mimic: fish A (*f* = 32 Hz, |θ_max_| = 25°), fish B (*f* = 40 Hz, |θ_max_| = 25°), fish C (*f* = 63 Hz, |θ_max_| = 30°), fish D (*f* = 75 Hz, |θ_max_| = 30°), and fish E (*f* = 83 Hz, |θ_max_| = 35°). The corresponding original data names from the work of van Leeuwen *et al.* (*21*) are summarized in table S7. Currently, the emulation is made in half period since not all the midline kinematics from the fish swimming are perfectly cyclic. The design parameters for each bioinspired robot are summarized in fig. S3 and table S3.

### Evaluation of the robot swimming performance

We use the average swimming speed of the CoM along the propulsion direction (denoted as ${\bar{\overset{\cdot }{w}}}_{x}$), the input power consumption (denoted as *P*_in_), and the CoT to quantitatively analyze the performance. Only the periods after the robots swim away from the launch stage (fig. S6) for at least three periods are processed for analyses. CoT is defined as locomotion-related energy consumption per unit distance and unit mass (*38*)

$$\text{CoT}=\frac{E_{\text{input}}}{\mathrm{dm}}$$ (1)

where *E*_input_ is the input energy for locomotion in three cyclic swimming periods, *d* is the covered distance along the propulsion direction, and *m* is the mass of the robot. The input energy equals to work done by the external magnetic torque, which can be computed as

$$\begin{array}{cc} E_{\text{input}} & =\int_{\theta_{\text{head}}(t_{1})}^{\theta_{\text{head}}(t_{1}+3T)}|T_{m}d\theta_{\text{head}}|=\\ & =\int_{t_{1}}^{t_{1}+3T}|m||B||\text{sin}(\alpha )||{\overset{\cdot }{\theta }}_{\text{head}}|\mathrm{dt} \end{array}$$ (2)

where *T*_m_ is the magnetic torque applied on the magnetic composite head of the robot, ***m*** is the magnetic moment of the head, ***B*** is the actuation magnetic flux density, α represents the angle between ***m*** and ***B***, and θ_head_ is the head pitch angle rotated by magnetic torque. Note that no matter the magnetic torque accelerates or deaccelerates the head, the input power is regarded as positive.

### Dynamic modeling of the fluid-structure interaction

The robots are simplified and modeled approximately as five-link swimmers (fig. S8A). The interaction between the robot and the surrounding fluid causes changes in body shape, leading to undulatory swimming. The dynamics can be modeled by (*39*–*42*)

$$J(\theta )\ddot{\theta }+C(\theta ){\overset{\cdot }{\theta }}^{2}=D\prime u_{\text{ela}}+u_{\text{mag}}+\tau_{e}+L\prime f_{e}$$ (3)

$$m\ddot{w}=E\prime f_{e}$$ (4)

where ***J***(**θ**) ∈ ***R***^5 × 5^ and ***C***(**θ**) ∈ ***R***^5 × 5^ are the matrices of the moment of inertia and centrifugal term, respectively, **θ** ∈ ***R***^5 × 1^ is the vector that contains counterclockwise angular displacement of the five links away from the *x* axis, ***u***_ela_ ∈ ***R***^4 × 1^ is the vector containing recovery torque on joints generated from the joint elasticity, ***u***_mag_ ∈ ***R***^5 × 1^ is the vector with the first entry as the magnetic torque on the head link and the rest entries are zero, ***f***_e_ ∈ ***R***^10 × 1^ and **τ**_e_ ∈ ***R***^5 × 1^ are the fluidic forces and torques acting on links, respectively, ***w*** ∈ ***R***^2 × 1^ is the displacement of the robot’s CoM on the plane of undulation, *m* is the mass of the robot, L is a projection matrix, and ***D*** and ***E*** are two constant matrices. We estimate the drag coefficients for fluidic forces and torques from the above dynamics equations and experiment data; then, we use these estimated coefficients to predict the swimming performances. For details and validation of the model, see note S4.

### PIV system

The PIV technique characterizes the fluid flow around the robot. We evenly seed the experiment tank with 1-μm polystyrene particles loaded with fluorochrome dye (1.1 ± 0.035 μm, Molecular Probes Inc.). These particles can be excited with a laser at 535-nm wavelength and emit fluorescence at 575 nm. The laser beam (1000 Hz, 527 nm) is expanded into a 0.5-mm-thick plane and projected along the plane of robot swimming. The particles are captured using a high-speed camera (M310, Phantom Inc.). A 570-nm high-pass lens filter is used to increase the contrast between the PIV particles and the background. The image sequences recorded are then processed in commercial software (DynamicStudio 2016a, Dantec Dynamics Inc.) to obtain velocity fields by applying the cross-correlation algorithm. A diagram combining the basic experiment setup and the PIV system is shown in fig. S6.

### Stiffness distribution–tunable SMP-based robot and its experimental setup

The synthesis method of the SMP material is referred from the work of Xie and Rousseau (*19*). The materials used for the synthesis are poly(bisphenol A-co-epichlorohydrin) glycidyl end-capped (PBGD), poly(propylene glycol) bis(2-aminopropyl ether) (Jeffamine D230), neopentyl glycol diglycidyl ether (NGDE), and poly(vinyl alcohol) (PVA) (Sigma-Aldrich). First, a square mold with a dimension of 1 cm by 1 cm by 50 μm (length by width by height) made by tapes is stuck on the glass substrate. Then 10 weight % PVA aqueous solution is poured into the mold and dried at 80°C for 30 min to form a sacrificial layer on the surface of the glass substrate. Meanwhile, 1 g of PBGD is melted by heating in an oven at 70°C for 20 min. Next, 200 μl of Jeffamine D230 and 250 μl of NGDE are added into the melted PBGD and stirred for 5 min. The resulting solution is spin-coated onto the above-prepared glass substrate at the speed of 800 rpm for 5 min. Last, after curing at 100°C for 1.5 hours in an oven and postcuring at 130°C for 1 hour, the SMP is immersed into deionized water for 6 hours to be released from the glass substrate and ready for use. These procedures produce the SMP with an effective thickness of 40 ± 10 μm.

Then, we integrate SMP to the robot to realize the stiffness adjustment capability, triggered by different water temperatures. The robot design parameters are summarized in table S4. Three SMP sheets are fixed at joints 2 to 4 by Ecoflex 00-10 (Smooth-On Inc.). The bending stiffness and Young’s modulus of the joints at different temperatures are estimated from the midline kinematics during cyclic swimming with different frequencies (see note S5). To reduce the robot’s effective density and the mutual friction with the tank bottom, we place a hollow 3D printed resin bubble (IP-Q, Nanoscribe GmbH) with the diameter *D*_b_ = 1 mm at the head.

To visually demonstrate the effects of *k*, we let the robot swim against the flowing flow. A custom water tank with an inner cross section of 10 mm by 10 mm is directly 3D printed (VeroClear and Objet260 Connex3, Stratasys Ltd.). The printed tank is sealed by gluing a transparent PMMA board on the top. A thermocouple tip is also sealed inside the tank to monitor the flow temperature at the acquisition rate of 1 Hz. The inlet of the tank is connected to a bucket of clear water through a peristaltic pump (L/S digital pump drives, Cole-Parmer Instrument Company). The outlet of the tank is directly connected to the water bucket. The water can then circulate through the above components, and the flow rate can be controlled. By adding ice or hot water into the bucket, the temperature of the water flowing into the tank can be adjusted. To visualize the flow, we add microparticles (fluorescent green polyethylene microspheres with diameters of 212 to 250 μm; Cospheric LLC) to the water during the video recording. It can be seen from movie S4 that the flow shows a noncontinuous behavior, and it is due to the nature of the peristaltic pump. The experiment environment–related parameters are summarized in table S6.

## SUPPLEMENTARY MATERIALS

Supplementary material for this article is available at http://advances.sciencemag.org/cgi/content/full/7/19/eabf7364/DC1
